# The Endotoxin Hypothesis of Parkinson's Disease

**DOI:** 10.1002/mds.29432

**Published:** 2023-05-08

**Authors:** Guy C. Brown, Marta Camacho, Caroline H. Williams‐Gray

**Affiliations:** ^1^ Department of Biochemistry University of Cambridge Cambridge UK; ^2^ Department of Clinical Neurosciences University of Cambridge Cambridge UK

**Keywords:** lipopolysaccharide, Parkinson's disease, endotoxin, inflammation, microglia, gut, neurodegeneration, neuroinflammation

## Abstract

The endotoxin hypothesis of Parkinson's disease (PD) is the idea that lipopolysaccharide (LPS) endotoxins contribute to the pathogenesis of this disorder. LPS endotoxins are found in, and released from, the outer membrane of Gram‐negative bacteria, for example in the gut. It is proposed that gut dysfunction in early PD leads to elevated LPS levels in the gut wall and blood, which promotes both α‐synuclein aggregation in the enteric neurons and a peripheral inflammatory response. Communication to the brain via circulating LPS and cytokines in the blood and/or the gut–brain axis leads to neuroinflammation and spreading of α‐synuclein pathology, exacerbating neurodegeneration in brainstem nuclei and loss of dopaminergic neurons in the substantia nigra, and manifesting in the clinical symptoms of PD. The evidence supporting this hypothesis includes: (1) gut dysfunction, permeability, and bacterial changes occur early in PD, (2) serum levels of LPS are increased in a proportion of PD patients, (3) LPS induces α‐synuclein expression, aggregation, and neurotoxicity, (4) LPS causes activation of peripheral monocytes leading to inflammatory cytokine production, and (5) blood LPS causes brain inflammation and specific loss of midbrain dopaminergic neurons, mediated by microglia. If the hypothesis is correct, then treatment options might include: (1) changing the gut microbiome, (2) reducing gut permeability, (3) reducing circulating LPS levels, or (4) blocking the response of immune cells and microglia to LPS. However, the hypothesis has a number of limitations and requires further testing, in particular whether reducing LPS levels can reduce PD incidence, progression, or severity. © 2023 The Authors. *Movement Disorders* published by Wiley Periodicals LLC on behalf of International Parkinson and Movement Disorder Society.

## Graphical abstract

The endotoxin hypothesis of Parkinson's disease (PD) proposes that gut lipopolysaccharide (LPS) promotes local α‐synuclein expression and aggregation, which then spreads via the vagus nerve to the brain, inducing PD neuronal loss together with neuroinflammation induced by blood LPS and peripheral inflammation.

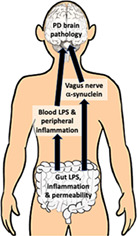

## 1 Introduction

Parkinson's disease (PD) is a common neurodegenerative disease affecting approximately 2% of people aged over 65 years in developed countries.^1^, ^2^, ^3^ It is characterized by progressive loss of midbrain dopaminergic neurons, and a movement disorder with slowness of movement, tremor, stiffness, and postural instability, plus a wide range of non‐motor symptoms.^2^, ^3^ A key neuropathological feature of PD is the presence of intraneuronal protein aggregates, known as Lewy bodies and Lewy neurites, the main component of which is fibrillar α‐synuclein, from the SNCA gene.^2^ α‐Synuclein aggregates are also found within enteric neurons in the gut and it has been proposed that spread can occur via the vagus nerve to the brain.^4^ About 5%–10% of PD cases are caused by a single genetic variant, with mutations in GBA and LRRK2 being the commonest genetic risk factors.^3^, ^5^ Common genetic variants also contributes to so‐called ‘idiopathic’ PD, but the overall heritable component of disease is estimated to be around 35%,^5^ hence environmental factors also contribute substantially to its pathogenesis. This review outlines the hypothesis that endotoxin is one such factor.

**FIG 1:**
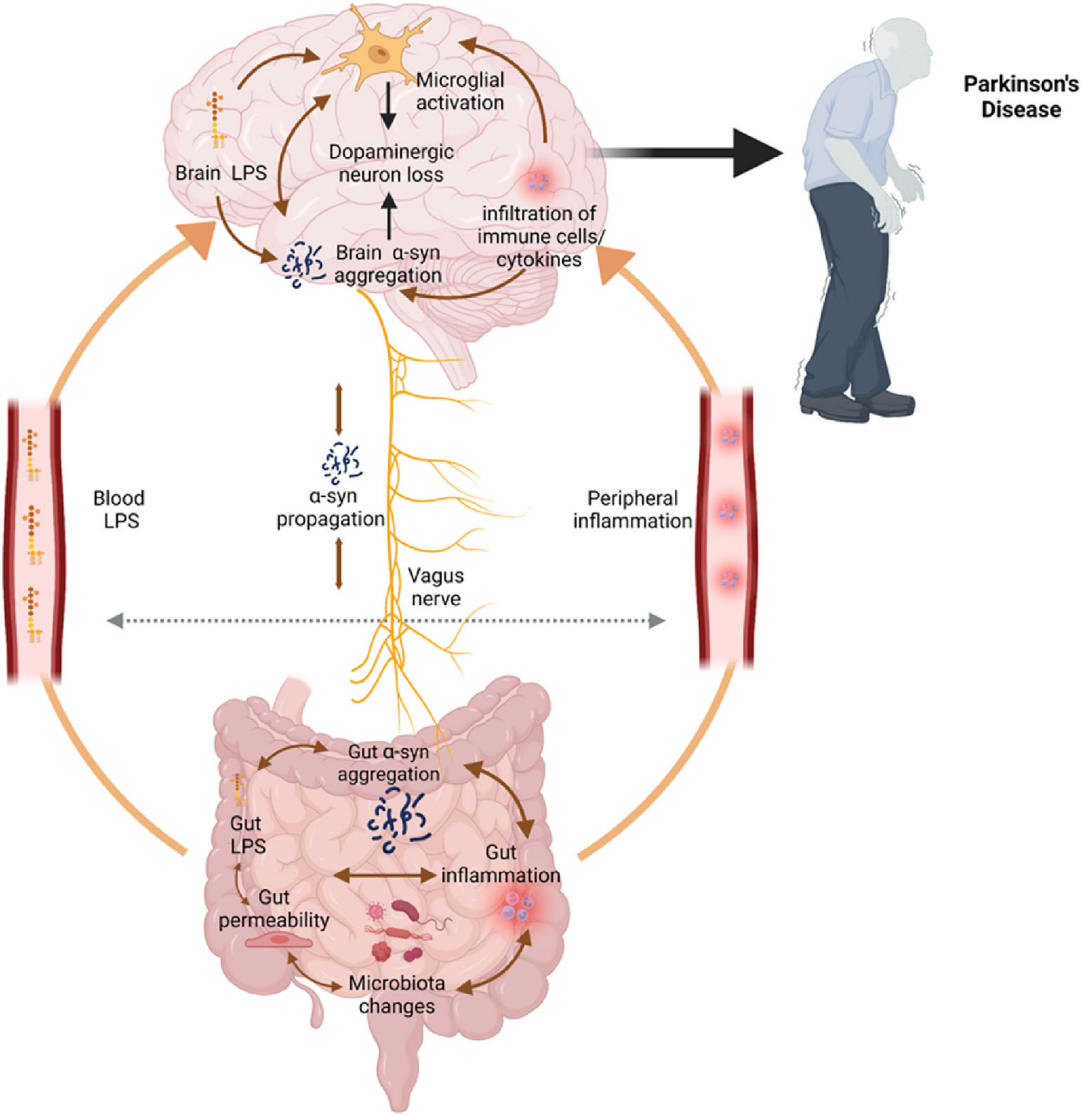
The endotoxin hypothesis of Parkinson's disease. Early microbiome changes and increased intestinal permeability elevate levels of lipopolysaccharide (LPS) in the gut wall. This promotes local inflammation, which induces α‐synuclein expression and aggregation locally in the gut, with propagation via the vagus nerve to brain. Gut inflammation and permeability also increase LPS levels and inflammation in the circulating blood, which promotes activation of microglia, α‐synuclein aggregation, and neurotoxicity in the brain. Figure created with BioRender.com. [Color figure can be viewed at wileyonlinelibrary.com]

A subset of PD patients in the pre‐motor stage of PD suffers from constipation, changes in the intestinal microbiome, and increased gut permeability.^6^, ^7^ This potentially facilitates translocation of endotoxins from the gut, where endotoxins are relatively benign, to the circulating bloodstream, where endotoxins induce an inflammatory response that can also affect the brain. Gut‐derived endotoxins may also promote α‐synuclein aggregation, and trigger systemic and brain inflammation, which exacerbates brain synucleinopathy and neuronal loss.^6^, ^7^, ^8^ A number of previous publications by us and others have linked endotoxin to PD,^6^, ^7^, ^8^, ^9^ but here we outline a specific hypothesis, together with the supporting evidence and its limitations, in order to facilitate assessment and testing of this theory (Fig. 1).

## 2 What Are Lipopolysaccharide Endotoxins and Where Do They Come From?

**FIG 2:**
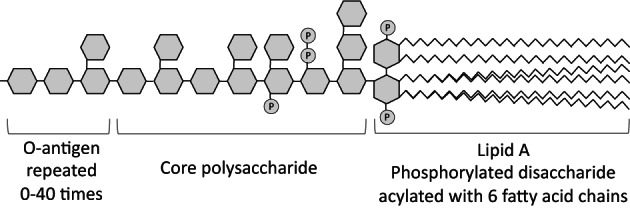
Structure of *Escherichia coli* lipopolysaccharide. The hexagons represent sugar monomers, circles with P represent phosphate groups, and wavy lines represent acyl chains (ie, fatty acids). The O‐antigen consists of a unit of four sugars repeated 0–40 times. Different bacteria have different endotoxin structures with additions or subtractions from the structure indicated.^10^

Endotoxins are bacterial components that when released are toxic to animals.^10^ Lipopolysaccharide (LPS) is the most abundant endotoxin, and the term endotoxin is commonly used interchangeably with LPS, so we will also use these terms interchangeably here. LPS consists of lipid A (usually a phosphorylated disaccharide attached to 6 acyl chains), connected to a ‘core’ (short sugar chain with modifications), connected to the O‐antigen (a long chain of sugars of variable length) (Fig. 2).^10^ The lipid A component of LPS constitutes much of the outer membrane of Gram‐negative bacteria, and the O‐antigen coats the surface of the bacterium. LPS is continually shed by live bacteria and also released when bacteria die.^10^ Once released from bacteria, LPS forms vesicles, because of the hydrophobic acyl chains, but may be transferred as a monomer to receptors by binding CD14 or lipopolysaccharide binding protein (LBP) in blood.^11^

Within the human body, the main sources of LPS are bacteria in the gut, skin, gums, lungs, or other epithelial surfaces, which may be increased by bacterial infections.^6^, ^7^, ^12^ The major source of endotoxin in healthy humans is gut‐resident Gram‐negative bacteria, including *Bacteroides fragilis* and *Escherichia coli*.^6^, ^7^ Limited quantities are thought to cross the intestinal wall, most of which is removed by the liver; however, small but significant levels of LPS are present in the blood of most humans.^6^, ^7^ LPS is present in food products^13^ and blood levels of LPS rise transiently after a high‐fat meal (‘metabolic endotoxemia^’^),^14^ so diet affects circulating LPS levels. LPS levels are also elevated in patients with liver disease, obesity, and intestinal permeability.^15^, ^16^, ^17^

Although the gut is the main source of LPS in the body, systemic bacterial infections can increase blood LPS levels and PD risk. For example, periodontitis (gum disease) increases blood LPS levels,^18^ and is associated with a 1.4‐fold increased risk of PD, and dental scaling to reduce infection also reduces PD risk.^18^, ^19^, ^20^ The main bacteria responsible for periodontitis is *Porphyromonas gingivalis*, which produces an inflammatory LPS found in blood and brain.^18^, ^21^, ^22^

## 3 Pathophysiology of LPS

Endotoxin can induce inflammation at very low concentrations via multiple receptors, including particularly TLR4 (toll‐like receptor 4) and its co‐receptor MD2.^10^, ^11^ CD14 and the LBP bind LPS and help transfer it to TLR4.^11^, ^23^, ^24^ Activation of TLR4 by endotoxin causes NF‐κB‐dependent transcriptional activation of hundreds of inflammatory genes, activation of the inflammasome, and the release of pro‐inflammatory cytokines such as TNFα, IL‐6, and pro‐IL‐1β.^25^, ^26^ Intracellular LPS can also directly activate caspase‐4 or caspase‐5 in humans (murine caspase‐11 in mice), which may then cleave and activate caspase‐1, which can cleave pro‐IL‐1β to IL‐1β.^27^, ^28^ In some cases, this may also cause cell death by pyroptosis, due to caspase‐1 or caspase‐11 cleaving and activating gasdermin D that permeabilizes the plasma membrane.^29^, ^30^ Endotoxin can also bind multiple scavenger receptors, activate complement via the alternative pathway, and directly activate complement receptor 3, which may contribute to the neurotoxicity.^31^, ^32^, ^33^

Different bacteria can have different variants of LPS with varying capacities to induce inflammation and toxicity.^10^ The lipid A component of LPS is sufficient to activate inflammation, but if the number of acyl chains on lipid A is reduced from 6 to 5 or 4, then its ability to activate TLR4 is greatly reduced.^34^ Indeed, LPS with 4 acyl chains (as produced by *Bacteroides dorei*) can inhibit inflammation, as it binds MD2/TLR4 without activating it, thus inhibiting activation by LPS with 6 acyl chains (as produced by *E. coli*).^34^ Thus, different bacteria in the gut, with different forms of LPS, can be pro‐ or anti‐inflammatory.

The innate immune system has a rapid and strong immune response to endotoxin because the presence of endotoxin is normally a sign of bacterial infection, which requires an early and robust immune response to prevent the infection spreading in the body. Thus, the immune response to endotoxin is normally beneficial. However, a chronic immune response to endotoxin over years may cause inflammatory changes in gut, body, and brain that precipitate PD, as proposed here.

## 4 Gut Bacteria as a Source of Endotoxin in PD

The gut lumen holds a large quantity of Gram‐negative bacteria containing LPS, which is potentially lethal if released into the blood.^7^, ^12^ The intestinal epithelium forms a barrier preventing endotoxin and bacteria entering the body and blood. A proportion of patients with early PD have increased intestinal permeability, informally called ‘leaky gut’.^7^, ^35^ A leaky gut may cause inflammation of the gut wall, partly because of the endotoxin leaking into the gut wall, and endotoxin can itself induce intestinal permeability and gut inflammation.^36^, ^37^, ^38^ Gut inflammation in turn promotes α‐synuclein expression and aggregation in the neurons of the gut in animal models (note, however, that the aggregation was found in an animal model overexpressing α‐synuclein).^39^ α‐Synuclein aggregation in the gut has been observed in colonic biopsies in early PD cases, and may contribute to gut dysfunction, through impacting on gut sensitivity and motility.^40^

Constipation is a common feature of early PD, prior to motor symptoms,^41^, ^42^ supporting the idea that pathological changes in the gut may be one of the earliest features of the disease, at least in a subset of patients. Around 50% of PD patients have constipation, versus about 18% in the general population; constipation increases the odds ratio of having PD by between 2‐ and 10‐fold^39^, ^43^, ^44^; and early constipation in PD has prognostic significance, predicting faster dementia onset.^45^

Several studies have found changes in the intestinal microbiome in PD patients that correlate with motor symptoms.^39^, ^46^, ^47^, ^48^ For example, Gorecki et al^38^ reported that the gut microbiome of non‐PD controls was 74% Clostridia (a benign Gram‐positive bacteria) and 19% Bacteroidia (producing a LPS that is anti‐inflammatory), whereas that of PD patients was 33% Gammaproteobacteria and 19% Verrucomicrobiae (both producing inflammatory LPS). Scheperjans et al^46^ found an increased proportion of LPS‐producing Gammaproteobacteria and Enterobacteriaceae in PD patients, and reduced Prevotellaceae, which correlated with increased intestinal permeability. Choi et al^49^ found that the Enterobacteriaceae bacterium *Proteus mirabilis* was increased in several mouse models of PD. They also found that oral *P. mirabilis* was sufficient to induce aggregation of α‐synuclein in gut and brain, as well as selective dopaminergic neuronal loss and motor deficits in mice, which was attributed to LPS from *P. mirabilis*. Yan et al^50^ also found a large increase in ratio of Gram‐negative to Gram‐positive bacteria in the gut of PD patients. A higher incidence of gut infections by Gram‐negative *Helicobacter pylori* and peptic ulcers has been reported in PD patients up to 10 years before motor symptoms,^51^, ^52^ and eradication of *H. pylori* with antibiotics improved PD symptoms.^52^, ^53^

Changes in the species of bacteria living in the intestinal lumen affect barrier permeability by regulating the tight junctions that link the epithelial cells together.^36^, ^54^, ^55^ Thus, it is possible that the changes in intestinal microbiome may initiate PD in the gut, potentially via endotoxin increasing intestinal permeability and inflammation, triggering α‐synuclein expression and aggregation in enteric neurons. However, it is also possible that changes in the gut microbiome arise as a consequence of other gut changes in PD, such as gut inflammation, enteric nervous system dysfunction, or reduced gut motility.

## 5 Blood Endotoxin Levels Are Elevated in PD

Blood endotoxin levels have been estimated in PD patients and age‐matched controls in four different studies listed in Table 1, using different methods. De Waal et al^56^ quantified binding of anti‐LPS antibody to platelet‐poor plasma and found a roughly 8‐fold increase in mean LPS level in PD patients versus controls. Loffredo et al^57^ quantified serum LPS levels using both sandwich enzyme‐linked immunosorbent assay (ELISA) (with anti‐LPS antibodies) and *Limulus* amoebocyte lysate (LAL) assay and found a roughly 2.4‐fold increase in mean LPS levels in PD patients. The LAL test measures the biological activity of LPS‐containing samples to induce coagulation of the blood cells of the horseshoe crab *Limulus*, and is quantified in endotoxin units (EU) of activity, where 1 EU roughly equates to 100 pg LPS, although this varies with the source of LPS. Our own study^8^ used the LAL assay and found an approximately 60% higher mean LPS level in serum of PD patients. Forsyth et al^35^ also used the LAL assay and found no significant change in serum LPS between PD patients and controls; however, they specifically excluded PD patients with constipation. Thus, the results of Forsyth et al^35^ lend support to the theory that elevated blood endotoxin levels in PD are linked to gut dysfunction.

**TABLE 1:** Studies quantifying lipopolysaccharide levels in serum or plasma of participants with Parkinson's disease and controls

Study	LPS in controls	LPS in PD	Method
Forsyth et al (2011)^35^	0.82 ± 0.21 EU/mL Mean ± SEM, N = 10	0.84 ± 0.13 EU/mL Mean ± SEM, N = 9	LAL assay in serum (excluded constipated patients)
De Waal et al (2018)^56^	0.51 ± 0.18 AU Mean ± SD, N = 11	3.9 ± 0.7 AU Mean ± SD, N = 11	Anti‐LPS antibody binding to platelet‐poor plasma
Loffredo et al (2020)^57^	12 ± 6 pg/mL Mean ± SD, N = 64	29 ± 5 pg/mL Mean ± SD, N = 8	LAL assay and sandwich ELISA in serum
Wijeyekoon et al (2020)^8^	1.20 ± 0.64 EU/mL Mean ± SD, N = 41	1.91 ± 1.66 EU/mL Mean ± SD, N = 41	LAL assay in serum

Note: Mean ± SD/SEM displayed accordingly.

Abbreviation: LPS, lipopolysaccharide; PD, Parkinson's disease; EU, endotoxin unit; LAL, *Limulus* amoebocyte lysate; SEM, standard error of the mean; N, number of people sampled; AU, arbitrary units; SD, standard deviation; ELISA, enzyme‐linked immunosorbent assay.

**FIG 3:**
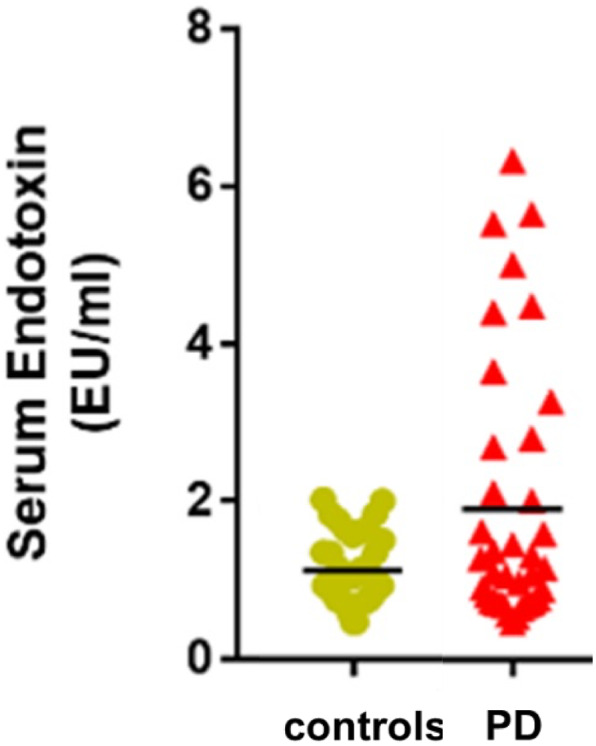
A proportion of Parkinson's disease (PD) patients have elevated endotoxin levels. Serum levels of endotoxin were measured by *Limulus* amoebocyte lysate (LAL) assay in 41 PD patients and 41 age‐matched controls. Each data point is the measured serum endotoxin level for one PD patient or control person. The black bars are the mean endotoxin levels. *P* = 0.023. EU, endotoxin unit. Adapted from Wijeyekoon et al (2020).^8^ [Color figure can be viewed at wileyonlinelibrary.com]

All these studies used a relatively small number of PD patients (N values in Table 1), the largest being our own^8^ with 41 PD patients and 41 age‐matched controls. In this study, about 25% of the PD patients had endotoxin levels higher than any of the controls; however, 70% of PD patients had normal levels of serum endotoxin (Fig. 3). This underlines the biological heterogeneity of PD and suggests that gut dysfunction and elevated serum endotoxin may be particularly relevant to disease pathogenesis in a subgroup of PD patients. In keeping with this, early gastrointestinal symptoms in PD are not universal, with around 30% of newly‐diagnosed PD patients reporting constipation.^58^ Our findings require validation, and longitudinal analysis of serum endotoxin levels in PD patients over days, months, and years would be of interest to determine whether endotoxin levels are elevated transiently or permanently, and whether levels correlate with symptoms and disease progression.

Despite these interesting data suggesting elevated LPS levels in PD, LPS quantification in human blood has many limitations. First, LPS blood concentrations are very low and may be below detection range in many commercially available assays. Potential contamination is also a significant issue during blood sampling (most commercially available blood collection tubes may contain traces of endotoxin), sample preparation, and assay testing.^59^ Salden and Bas recommend immediate cooling of blood samples to 0°C and immediate centrifuging of samples.^59^ Blood coagulation results in additional loss of endotoxin activity, so plasma is preferable to serum and collection tubes should have low concentrations of heparin.^59^, ^60^ Technical aspects of the different assays, comparisons between assay sensitivity, as well as differential sample preparation methods, have been described in detail elsewhere.^60^, ^61^

Because of the difficulties of measuring LPS in blood samples and the short half‐life of LPS in blood, indirect markers of systemic endotoxin levels have also been used. Lipopolysaccharide‐binding protein (LBP) can neutralize endotoxin and help remove it from blood, hence lower levels are suggestive of increased endotoxin exposure or availability.^62^ Multiple studies have reported reduced serum or plasma LBP levels in PD patients compared with controls,^35^, ^47^, ^63^, ^64^, ^65^ and lower levels have also been found to be linked to increased risk of PD.^65^

## 6 Peripheral Endotoxin Can Induce PD‐Like Pathology

Injection of LPS into the peritoneum of mice or rats induces chronic neuroinflammation, progressive nigrostriatal pathology, motor deficits, and specific degeneration of dopaminergic neurons in the substantia nigra, and this is now used as a mouse model of PD.^33^, ^66^, ^67^, ^68^, ^69^, ^70^, ^71^ This surprising finding is of obvious relevance to PD, as it indicates that elevated LPS in the periphery (as found in a proportion of PD patients) is sufficient to induce the specific neuronal loss causing PD motor symptoms.

In humans, there is a single case study of a laboratory worker who developed parkinsonism 3 weeks after accidental exposure to 10 μg endotoxin through an open wound.^72^ The LPS caused chronic inflammation in the nervous system, and damage to the substantia nigra, with bradykinesia, rigidity, and tremor.^72^, ^73^ This amount (10 μg) of endotoxin distributed through the whole body is equivalent to 140 pg/mL, which compares to 30 pg/mL found in the serum of PD patients.^57^

Injection of LPS into healthy humans induces a number of symptoms (for a few hours), including pain, fatigue, increased sleep, low appetite, and depressive symptoms.^74^, ^75^ Some of these overlap with the non‐motor symptoms commonly reported in people with PD.^38^ Hence, it is possible that LPS is a contributory factor to these non‐motor symptoms in PD. However, the pathophysiological basis of these non‐motor features is multifactorial, and the sickness behavior induced by LPS can be induced by many other factors.

## 7 Endotoxin Can Synergize with α‐Synuclein in Mice

In model systems, endotoxin can interact or synergize with α‐synuclein to induce PD pathology in a variety of ways. For example, injection of endotoxin into the peritoneum of mice induced α‐synuclein expression and phosphorylation in the colon, and increased gut permeability.^37^ Endotoxin can also induce α‐synuclein to fibrillize into novel forms that seed further self‐sustaining fibrillization.^76^, ^77^ Peripheral endotoxin increased blood–brain barrier permeability and increased uptake of α‐synuclein into the brains of mice.^78^ Peripheral injection of endotoxin into control mice and A53T α‐synuclein transgenic mice caused similar acute neuroinflammation, but only the transgenic mice then developed persistent neuroinflammation, α‐synuclein aggregation, Lewy bodies, and progressive loss of dopaminergic neurons in the substantia nigra.^79^ Similarly, mice expressing human A53T α‐synuclein or injected with a single dose of LPS had no loss of dopaminergic neurons in the substantia nigra 13 months later, but the combination induced substantial neuronal loss.^80^ In α‐synuclein overexpressing mice, oral administration of LPS induced the early onset of motor symptoms.^38^ This suggests a dual‐hit hypothesis for PD: elevated endotoxin plus aggregable α‐synuclein may drive the neuronal loss of PD.

## 8 Endotoxin May be Relevant in Genetic Forms of PD

About 5%–10% of PD is monogenic, that is, due to mutation in a single gene, with at least 14 such genes being identified to date, including *GBA*, *LRRK2*, *Parkin*, *DJ1*, *PINK1*, and *SNCA*.^5^ We have noted earlier that endotoxin activates the SNCA gene to express α‐synuclein, and synergizes with α‐synuclein to induce neuropathology. Similarly, mutant *LRRK2* potentiates dopaminergic neuronal loss in the substantia nigra of mice induced by peripheral LPS.^81^ *GBA* mutations increase the inflammatory response of macrophages to LPS, measured as cytokine release.^82^, ^83^ Similarly, *Parkin* knockout mice have increased LPS‐induced neuroinflammation, dopaminergic neuronal loss, and motor deficits,^68^ and mutant or knockout *DJ1* increases LPS‐induced microglial activation and loss of dopaminergic neurons in culture and in vivo.^84^, ^85^ So, in general, genetic mutations linked to PD are associated with an increased inflammatory response to LPS, promoting neuronal loss. Thus, the endotoxin hypothesis of PD may be of relevance for both genetic and idiopathic forms of PD.

## 9 Endotoxin Induces Systemic Inflammation that May Contribute to PD

LPS is a potent activator of monocytes, binding via TLR4 to trigger downstream activation of NF‐κB and IRF transcription factors and production of inflammatory cytokines and chemokines. In healthy human volunteers, intravenous injection of LPS induces serum TNFα, IL‐6, IL‐8, and IL‐10, followed by neuroinflammation.^74^, ^75^ In vitro studies have indicated that monocytes from PD patients have an increased response to LPS stimulation that correlates with disease severity,^86^ although this is not a universal finding and may vary according to disease stage and sex.^87^ In addition, the LPS‐receptor TLR4 is upregulated in blood‐derived monocytes in PD, as well as in the gut submucosa,^47^ suggesting that monocytes may be primed to respond to LPS.

There is growing evidence that innate immunity and inflammation contribute to the pathophysiology of PD.^88^, ^89^, ^90^ Levels of inflammatory cytokines are elevated in the serum in PD,^91^ and a more pro‐inflammatory profile of immune markers in the blood is associated with more rapid disease progression.^92^ Monocytes are a major source of these cytokines, and their phenotype is altered in people with PD, with an elevated proportion of classical monocytes which are highly phagocytic and inflammatory, and increased expression of monocyte receptors involved in cell activation and migration.^8^, ^86^ These monocyte changes are most marked in those at higher risk of dementia, suggesting that innate immune activation may contribute to more rapid disease progression.^8^ Changes in monocyte subsets have been reported prior to PD diagnosis in ‘at risk’ populations, providing support for the hypothesis that the innate immune response contributes to disease onset.^93^ In addition, activation of inflammatory macrophages within the gut in mice promotes pathological α‐synuclein aggregation in enteric neurons with propagation via the vagus nerve.^94^

Peripheral TNFα, IL‐1β, and LPS can each induce brain inflammation, including release of cytokines and chemokines within the brain in mice.^95^ The mechanisms of peripheral–central immune crosstalk in PD are still not fully established but an endotoxin‐driven peripheral inflammatory response may influence brain pathology via infiltration of immune cells via the choroid plexus and/or passage of cytokines across the blood brain barrier (BBB).^96^

## 10 Endotoxin May Enter the Brain and Induce Neurodegeneration Mediated by Microglia

LPS and related bacterial proteins have been detected in human healthy brains and shown to be increased in brains from Alzheimer's disease patients.^97^ It is not clear how endotoxin in the blood might enter the brain, but high levels of endotoxin in blood can increase blood–brain permeability,^97^, ^98^ potentially allowing endotoxin and other inflammogens into the brain. In more physiological conditions, endotoxin may enter the brain via lipoprotein transport mechanisms, at least in rats.^99^ Sustained brain inflammation in response to blood endotoxin requires brain TLR4, and is independent of blood cytokines, suggesting that endotoxin enters the brain and activates TLR4 there to sustain neuroinflammation, at least with acute LPS administration in mice.^100^ Multiple studies have demonstrated that TLR4 expression is increased in the PD post‐mortem brain, suggesting the PD brain may be more sensitive to LPS.^101^, ^102^, ^103^

Direct stereotaxic injection of endotoxin into the substantia nigra of mice induces microglial activation and degeneration of the dopaminergic neurons of this area.^104^, ^105^, ^106^ Similarly, chronic peripheral injections of LPS result in a rather specific loss of midbrain dopaminergic neurons in mice, mediated by microglia.^33^, ^68^, ^69^, ^70^, ^71^

Microglia are brain macrophages, the main mediators of innate immunity and inflammation in the brain, and microglia are known to become activated in the substantia nigra of PD patients.^107^, ^108^, ^109^ Endotoxin can directly induce inflammatory activation of microglia, and endotoxin‐induced activation of microglia can cause death or loss of dopaminergic neurons in culture,^110^ as well as in mouse substantia nigra.^33^, ^68^, ^69^, ^70^, ^71^ For example, injection of endotoxin into the peritoneum of mice caused acute and permanent activation of brain microglia, and loss of dopaminergic neurons in the substantia nigra 10 months later.^66^, ^69^ In healthy human volunteers, intravenous injection of 1 ng LPS/kg caused a robust microglial activation in most areas of the brain measured by positron emission tomography (PET) imaging of a peripheral benzodiazepine receptor (PBR) ligand 3 hours after LPS injection.^75^

**FIG 4:**
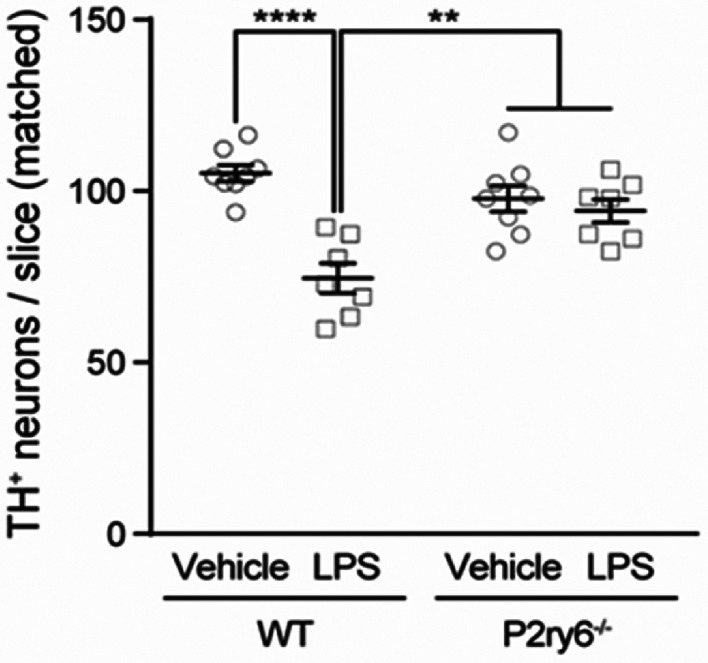
Peripherally administered endotoxin causes specific loss of dopaminergic (TH^+^) neurons in the substantia nigra of mice, prevented by knockout of the P2Y6 receptor (P2ry6^−/−^) required for microglial phagocytosis of neurons. LPS, lipopolysaccharide; WT, wildtype. Adapted from Milde et al (2021).^71^

Endotoxin‐activated microglia might induce neurodegeneration by multiple means. In glial‐neuronal co‐cultures, endotoxin can induce neuronal loss, and eliminating microglia prevents this neuronal loss.^110^ In the presence of IFNγ, LPS can induce inducible nitric oxide synthase (iNOS) in glia and the nitric oxide can then kill neurons,^111^ particularly if this is combined with either hypoxia or superoxide.^112^, ^113^ However, endotoxin alone causes no neuronal necrosis or apoptosis, but rather microglial phagocytosis of live neurons, resulting in death of the neurons as a result of the phagocytosis.^105^, ^114^ Engulfment seems to require uridine diphosphate (UDP) release from the stressed neurons, which then activates the P2Y6 receptor on microglia, triggering the microglial phagocytosis of the stressed‐but‐viable neurons.^115^ Thus, this neuronal loss can be prevented by blocking the P2Y6 receptor.^71^, ^115^ For example, peripheral endotoxin‐induced loss of dopaminergic neurons in mouse substantia nigra was prevented in P2Y6 receptor knockout mice (Fig. 4).^71^ Injection of LPS into the striatum of rats also induced neuronal loss that was prevented by a P2Y6 receptor inhibitor.^115^ Peripheral endotoxin can also activate the classical complement system in brain, which activates microglial phagocytosis, resulting in neuronal loss that can be prevented in complement C3‐deficent mice.^33^

## 11 Potential Treatments Based on the Endotoxin Hypothesis

If LPS contributes to PD, then a number of possible therapeutic strategies could be considered for further evaluation in clinical trials as outlined below. However, if LPS is elevated in only a subset of PD, patient stratification would be essential for targeting the most appropriate patients. Monitoring of blood LPS levels in such trials would provide important validation of the mechanistic principle, alongside clinical efficacy measures.

(1) Changing the gut microbiome. Manipulating the gut bacterial profile to reduce inflammatory endotoxin‐producing species could be achieved with specific antibiotics, or oral bacteria, or fecal microbiota transplant (FMT).^116^ To date, clinical trials of antibiotic treatments for PD have not supported their use.^52^ It should also be noted that specific strains of bacteria degrade L‐dopa in the gut^117^ and affect dopamine availability hence some antibiotics might enhance levodopa absorption, thus complicating interpretation of clinical effects.

(2) Reducing gut permeability. This might also be achieved by manipulating the gut microbiome through upregulation of mucin‐producing bacteria or reduction of mucin‐degrading bacteria.^118^ Non‐steroidal anti‐inflammatory drug (NSAID) use is paradoxically associated with gut inflammation.^119^ Azathioprine reduced gut permeability in patients with ulcerative colitis, likely via reducing gut inflammation.^120^ Anti‐TNFα antibodies reduce gut inflammation and permeability in patients with Crohn's disease, suggesting that the inflammation is responsible for the permeability increase.^121^ Elemental diet also reverses the gut permeability increase seen in Crohn's disease.^122^ Metformin reduces gut permeability in mice,^123^ and has other effects which may potentially be beneficial in PD.^124^

(3) Reducing LPS levels in blood. This might be achievable via vaccination against endotoxin. Vaccination of animals with detoxified LPS can induce anti‐LPS antibodies, which protects these animals from subsequent Gram‐negative sepsis.^125^, ^126^ Immunization of cows with detoxified LPS can induce anti‐LPS antibodies in colostrum, which when fed to rats can reduce blood LPS levels.^127^

(4) Blocking LPS receptors. Candesartan, an existing drug licensed for the treatment of hypertension, has been shown to reduce TLR4 expression and activity, is expected to cross the BBB, and has a good safety profile, making it an attractive candidate for repurposing in Parkinson's disease.^124^, ^128^ TAK242 is a potent small molecule inhibitor of TLR4, which has previously been used in a clinical trial for treatment of sepsis and shown to be well tolerated in the short term.^129^

(5) Blocking the microglial response to LPS. In addition to blocking TLR4, possible strategies to reduce the microglial response to endotoxin could include blocking complement receptor 3, the P2Y6 receptor, or the inflammasome.^33^, ^71^

## 12 Limitations of the Hypothesis and LPS Measurements

Rodent models have been used to provide evidence supporting the endotoxin hypothesis. However, mice are less sensitive to endotoxin than humans, and therefore higher levels of endotoxin are injected into mice than humans can tolerate, thus the physiological relevance of these models is unclear.^74^ Furthermore, the response to endotoxin depends on the time course and pattern of administration as prior exposure to endotoxin can either sensitize or desensitize to subsequent exposure.^130^ Indeed, low dose LPS has been found to be neuroprotective in some animal models of neurodegeneration.^131^

Elevated blood LPS is also not specific to PD, but rather is found in multiple pathologies, including sepsis, liver disease, periodontitis, amyotrophic lateral sclerosis, and Alzheimer's disease.^9^ So, it cannot be true that elevated blood LPS is sufficient to induce PD. Thus, some form of dual‐hit hypothesis is required, for example, LPS plus α‐synuclein expression/aggregation, or LPS plus a relevant mutation in a PD‐associated gene, or LPS plus increased interferon expression. This is related to the idea that neurodegeneration primes the brain for an excessive LPS response, so that an episode of endotoxemia may exacerbate an otherwise‐independent neurodegenerative process.^132^, ^133^ Alternatively, prior exposure to LPS may prime the brain to a subsequent neurodegenerative process.^69^ LPS might also induce PD synergistically with environmental toxicants, such as pesticides or heavy metals, for example by LPS inducing expression of components and substrates of the inflammasome, which is then activated by such toxicants.^134^

It also appears to be true that elevated blood LPS is not required for PD, as we found that serum LPS was only elevated in a proportion (about 30%) of PD patients,^8^ and LPS levels were not elevated in PD patients without constipation.^35^ However, LPS measurements in PD have been limited by their cross‐sectional nature with measurement at a single timepoint. Whereas, in utero exposure of mice embryos to LPS results in loss of dopaminergic neurons in substantia nigra postnatally,^135^ and a single intraperitoneal injection of LPS in 2‐month‐old mice results in loss of dopaminergic neurons in the substantia nigra 10 months later.^69^ This raises the possibility that LPS exposure as a result of episodic infections or gut dysfunction may result in PD sometime later, without blood LPS levels being elevated at intermediate times.

The reproducibility and changes of LPS levels in blood (measured longitudinally in the same patients) needs to be tested in the short term (hours to days), and long term (years), and related to symptoms (eg, gut function) and clinical disease progression. It would also be useful to measure LPS levels in the prodromal stages of PD (eg, in cohorts with rapid eye movement [REM] Sleep Behavior Disorder [RBD]), alongside evaluation of gut function and PD conversion risk.

Measurement of LPS levels in human biosamples is technically challenging, with commercial kits for using LAL assays or sandwich ELISA often not being sufficiently sensitive for the levels found in human blood.^136^ Furthermore, there are several potential confounding factors. For example, albumin in serum can interfere with the LAL.^137^ Blood coagulation, required for separation of serum, can also lead to trapping of endotoxin, thus LPS quantification in plasma may be preferable and LPS levels in plasma are higher than in serum.^138^ Diet affects LPS‐producing bacteria in the diet, and blood endotoxin levels rise after a high fat diet.^14^ Antibiotic use leading to bacterial death may cause LPS to be shed into the bloodstream.^139^ Liver damage may also impact on LPS measurements given that the liver is the major site of endotoxin removal.^13^, ^140^ Vaccines contain endotoxin as an adjuvant, but they often contain aluminum hydroxide that interferes with the LAL assays, resulting in false‐negatives.^13^ We recommend the following for future studies measuring LPS levels in PD cohorts:

1. Endotoxin quantification should be ideally be performed in plasma samples
2. Endotoxin measurements should be done in fasted individuals
3. Intercurrent infections should be recorded
4. Blood collection should be done at least 1 week after latest vaccination
5. Blood collection should be avoided during periods of antibiotic use
6. Samples should be immediately cooled to 0°C and centrifuged.

## 13 Conclusions and Key Tests of the Endotoxin Hypothesis

The endotoxin hypothesis of PD suggests that elevated LPS levels contribute to the pathogenesis of PD. Gut dysfunction and a leaky gut may allow LPS into the gut wall, promoting local α‐synuclein expression and aggregation, which then spreads via the vagus nerve to the brain. Increased intestinal permeability may also lead to elevated LPS in the blood, which activates the peripheral innate immune system as well as brain microglia, and promotes α‐synuclein pathology in the brain, and loss of dopaminergic neurons in the substantia nigra. Given the clinical and biological heterogeneity of PD, these endotoxin‐related mechanisms may not be a universal feature of the disease, but may be highly relevant in a subset of patients.

Multiple variants of the endotoxin hypothesis are possible, but the key test of all these hypotheses is whether reducing endotoxin in those PD patients with elevated endotoxin can reduce the rate of disease progression. Such a trial would require selection of patients according to their baseline endotoxin levels. As an initial step, studies are needed to quantify LPS and its associated markers longitudinally in large PD cohorts as well as prodromal cohorts to determine the relationship between endotoxin levels, disease development, and progression.

## Author Roles

All authors participated in the design, drafting, and editing of the manuscript.

## Data Availability

Only publicly available data is evaluated in this manuscript.
